# COVID-19: Looking Into the Overlooked

**DOI:** 10.3389/fmolb.2020.00165

**Published:** 2020-08-11

**Authors:** Fernanda Cristina Petersen, Ulf Reidar Dahle, Belinda Nicolau, Climent Casals-Pascual

**Affiliations:** ^1^University of Oslo, Oslo, Norway; ^2^Centre for Antimicrobial Resistance, Norwegian Institute of Public Health, Oslo, Norway; ^3^Faculty of Dentistry, McGill University, Montreal, QC, Canada; ^4^Laboratorio de Microbiología, Centro de Diagnóstico Biomédico, Hospital Clínic de Barcelona, Barcelona, Spain; ^5^Hospital Clínic de Barcelona, Barcelona, Spain

**Keywords:** COVID- 19, microbiome, antibiotic resistance, SARS-CoV- 2, biomarker

Viral infections have plagued humanity since the beginning of time causing numerous deaths through specific pathogenic events directly related to the virus and indirectly, through secondary infections accompanying or following the viral episode. Highly pathogenic bacteria, such as *Neisseria meningitidis, Streptococcus pneumoniae*, and *Haemophilus influenzae* can overgrow during viral infections, and cause severe bacterial infections. During the 1918 Spanish influenza pandemic, for instance, the majority of influenza fatalities were likely caused by secondary pneumococcal pneumonia (Morens et al., 2008), but we were not aware of it until 2008, when tissue biopsy investigations revealed this important association. Secondary bacterial infections also accounted for 25–50% of deaths during the 2009 H1N1 influenza pandemic (Macintyre et al., 2018). In this instance, the association with secondary infections, particularly with *S. pneumoniae*, was acknowledged only almost 10 years after the pandemic.

In the current COVID-19 pandemic, the role of co-infectiveness remains unclear. At the peak of the pandemic, broad-spectrum antibiotics have been administered to the majority of patients admitted with COVID-19 to hospital to prevent secondary infections but also, some antibiotics, like teicoplanin, have been used due to their alleged antiviral properties. In an early report, secondary infections were detected in 1 out of 7 patients. Among those that died, 50% had secondary infections (Zhou F. et al., 2020). Other reports indicate that bacterial infections were documented in <10% of COVID-19 patients (Zhou P. et al., 2020). In light of this uncertainty, antibiotic use has been reported to be as high as 74% among patients with COVID-19 who were admitted to ICUs (Cox et al., 2020). Some infections may have been caused by changes in colonization resistance, others due to increase use of corticosteroids, parenteral nutrition or just intravenous catheter infections. The administration of broad-spectrum antibiotics in the absence of high suspicion or documented infection challenges the current dogma of antimicrobial stewardship. This problem is further compounded by the fact that acute respiratory syndromes due to infection or non-infection are notoriously difficult to discriminate. However, the risk and potential severity of secondary infections associated with COVID-19, particularly in overcrowded clinical settings and in immune-compromised patients, has in many cases reduced the compliance with the local prescription practice guidelines.

A quick look at host factors may now be more relevant than ever. As the first wave of the pandemic is tailing off in Europe, we have gathered substantial and actionable evidence that suggests that both severity and mortality associated with COVID-19 is due to host factors, ranging from endothelial and coagulation disturbances leading to the formation of micro thrombi to a major dysregulation of the host immunity and inflammatory response. Here, a recent study has shown significant survival benefits of corticosteroids administration to patients with severe COVID-19 infection.

How is the host contributing to the severity of COVID-19? It was a matter of time that a genome wide analysis study (GWAS) showed some association with disease severity. Indeed, we have recently learnt that particular blood groups (group A) may be linked to respiratory failure in COVID-19 patients (Ellinghaus et al., 2020). However, with a moderate increase severity risk (odds ratio: 1.45 with respect to other blood groups), it is unlikely that this association explain all the variability of disease severity. Here we should not forget that one of the major contributors that regulate individual immune/inflammatory responses is the human microbiome. In particular, the gut microbiome may influence directly and indirectly the immune response generated. Accordingly, the lymphocyte populations may adopt a more pro-inflammatory phenotype when microbiome diversity is reduced, as typically observed in old age patients (Figure 1) and recently reviewed by Dhar and Mohanty (2020).

**Figure 1:**
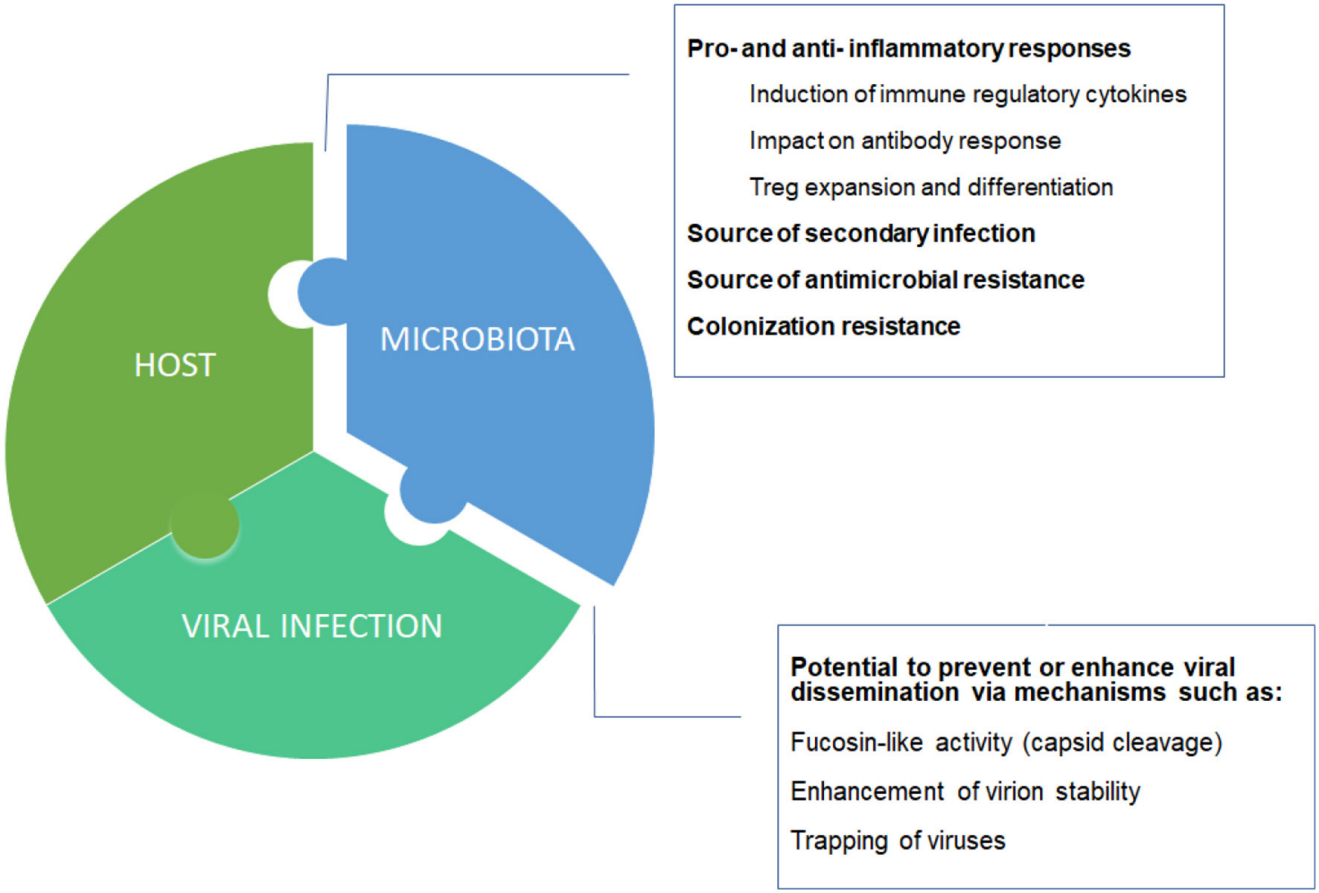
Host-microbiota-virus triumvirate. Individual microbiome taxonomic profile and metabolic capacities may affect the balance between pro- and anti- inflammatory responses, and between suppression or enhancement of viral dissemination.

Broad spectrum antibiotics are well-known to promote major disturbances in the microbiome. For critically ill patients, secondary infections and lack of response to antibiotics may be all that is necessary to shift the balance toward irreversible clinical deterioration and death. Also for some patients, antibiotics may do more harm than good. At least two of the antibiotics that are being used for treatment or prophylaxis in COVID-19 patients, ceftriaxone and teicoplanin (Martinez, 2020), have been known since the 1990s to promote a substantial increase in the population of resistant enterococci in the gut microbiota (Meijer-Severs et al., 1990; Van Der Auwera et al., 1996). Azithromycin is another antibiotic that, like teicoplanin, has been repurposed for use in COVID-19 patients due to its possible anti-SARS-CoV2 activity. Although there are few studies focusing on microbiota disturbances by azithromycin in adults, there are reports of macrolide resistance enrichment and reduction in diversity among patients with asthma (Taylor et al., 2019). Examples of promotion and suppression of viral infections in combination with antibiotics have been studied mostly in animal models. In humans, seminal studies have shown the impact on antibody response by approaches using antibiotic depletion of the microbiota in combination with live viral vaccines (Harris et al., 2018; Hagan et al., 2019).

The broad use of antibiotics during COVID-19 is *per se* an important reason to monitor changes in the microbiota of the respiratory or gastrointestinal tract. Moreover, in COVID-19, there are a number of striking observations in the course of the disease that we cannot yet explain, like healthy patients with minor or no co-morbidities that suddenly become irreversibly ill (Team, 2020). There are also more and more reports of patients cured with negative PCRs and positive antibodies that later on develop severe thrombotic events with reactivation of the virus (Oxley et al., 2020). Inflammatory responses are exacerbated in several cases, as indicated by cytokine storms. Understanding the link between microbiome, immunity and inflammatory response in SARS-CoV-2 infected patients could help explaining such unexpected outcomes. A plethora of mechanisms by which the gut microbiota modulates the immune system have been described in the literature, and reviewed in several articles (Maynard et al., 2012; Honda and Littman, 2016; Levy et al., 2016). One relevant example are short chain fatty acids provided by the gut microbiota, which have a critical role in promoting expansion and differentiation of regulatory T cells (Tregs), thus impacting maintenance of immune homeostasis (Tanoue et al., 2016). In the airways, Tregs induced by short chain fatty acids contribute among other effects, to suppression of lung inflammation (Trompette et al., 2014). Both pro- and anti- inflammatory stimulation, and responses promoting or suppressing viral infections have been reported as directly modulated by members of the microbiota (Li et al., 2019). Of note, old age and comorbidities such as diabetes and cardiovascular diseases, which are linked to COVID-19 severity and mortality are also both associated with dysbiotic states (Peterson et al., 2015; Yang et al., 2015; Li et al., 2017).

Quantifying microbiota-host imbalances would have the potential to yield novel diagnostics for risk stratification and improved clinical management. However, implementing such clinical tools within a reasonable time frame depends on collecting this information from patients now. International consortia and multicenter studies have in record time organized initiatives to create and explore existing biobanks for COVID-19, as to understand the diversity in disease manifestations. Several risk indexes are created or combined with those commonly used in ICUs, and numerous initiatives to identify human genetic factors, antibody responses, and viral changes are underway, with virtually no mention on the microbiota. After two decades of major milestone discoveries in the field, this is somehow surprising. So far, search for COVID-19 biobank initiatives at Biobanks Europe (https://www.bbmri-eric.eu) do not retrieve any hits for fecal samples. Actually, as of May 27th 2020, only seven in 1706 registries of COVID-19 studies in the ClinicalTrials.gov refers to microbiome or microbiota as an outcome measurement.

**“The art of being wise is the art of knowing what to overlook”- William James, 1890**

Focusing either on the host or on the virus during pandemics may be the wise way to go when resources are scarce and priorities must be established. On the other hand, the history of human infections, and the accumulated knowledge on the interplay of the human microbiota and the immune system indicate that we may be overlooking one of the three core elements in the present COVID-19 pandemic. With COVID-19 varying in severity from asymptomatic to lethal, the microbiota could provide valuable biomarkers to predict which individuals are most at risk of suffering severe disease.

## Author Contributions

FP wrote the paper with inputs from all authors. All authors conceived the presented ideas and approved the final manuscript.

## Conflict of Interest

The authors declare that the research was conducted in the absence of any commercial or financial relationships that could be construed as a potential conflict of interest.
